# Evidence of Impaired Brain Activity Balance after Passive Sensorimotor Stimulation in Multiple Sclerosis

**DOI:** 10.1371/journal.pone.0065315

**Published:** 2013-06-14

**Authors:** Nikolaos Petsas, Emanuele Tinelli, Delia Lenzi, Valentina Tomassini, Emilia Sbardella, Francesca Tona, Eytan Raz, Valter Nucciarelli, Carlo Pozzilli, Patrizia Pantano

**Affiliations:** 1 Department of Neurology and Psychiatry, Sapienza University of Rome, Rome, Italy; 2 Department of Psychology, Sapienza University of Rome, Rome, Italy; 3 Institute of Psychological Medicine and Clinical Neurosciences, Cardiff University School of Medicine, Cardiff, United Kingdom; 4 Santa Lucia Foundation, Rome, Italy; University of Utah School of Medicine, United States of America

## Abstract

**Objectives:**

Examination of sensorimotor activation alone in multiple sclerosis (MS) patients may not yield a comprehensive view of cerebral response to task stimulation. Additional information may be obtained by examining the negative BOLD response (deactivation). Aim of this work was to characterize activation and deactivation patterns during passive hand movements in MS patients.

**Methods:**

13 relapsing remitting-MS patients (RRMS), 18 secondary progressive-MS patients (SPMS) and 15 healthy controls (HC) underwent an fMRI study during passive right-hand movements. Activation and deactivation contrasts in the three groups were entered into ANOVA, age and gender corrected. Post-hoc analysis was performed with one-sample and two-sample t-tests. For each patient we obtained lesion volume (LV) from both T1- and T2-weighted images.

**Results:**

Activations showed a progressive extension to the ipsilateral brain hemisphere according to the group and the clinical form (HC<RRMS<SPMS). Significant deactivation of the ipsilateral cortical sensorimotor areas was reduced in both patient groups with respect to HC. Deactivation of posterior cortical areas belonging to the default mode network (DMN), was increased in RRMS, but not in SPMS, with respect to HC. The amount of activation in the contralateral sensorimotor cortex was significantly correlated with that of deactivation in the DMN in HC and RRMS, but not in SPMS. Both increased activation and decreased deactivation patterns correlated with LV.

**Conclusion:**

In RRMS patients, increased cortical activation was associated with increased deactivation of the posterior cortex suggesting a greater resting-state activity in the DMN, probably aimed at facilitating sensorimotor circuit engagement during task performance. In SPMS the coupling between increased sensorimotor activation/increased DMN deactivation was not observed suggesting disorganization between anticorrelated functional networks as a consequence of a higher level of disconnection.

## Introduction

The motor function of the nervous system has been extensively investigated in patients with MS by means of functional magnetic resonance imaging (fMRI). Several studies have reported that brain activation patterns in MS patients during an active hand movement are different from those of healthy subjects and are aimed, at least in part, at maintaining normal function [1], [2]. In particular, functional cortical changes occur in the early phase of disease [3], [4], are related to clinical symptoms and/or disability [3], [5]–[8], depend on lesion burden and/or ultra-structural damage [3], [5], [9]–[11], tend to change over time [12], [13] and may be modified by drugs or motor training [14]–[16].

Although these studies have largely increased our knowledge on brain plasticity in MS, we consider that examination of the positive BOLD response (activation) alone may not yield a comprehensive view of cerebral response to task stimulation. Additional information may be obtained by examining the negative BOLD response (deactivation) that indicates a decreased signal during task execution compared with the control condition.

Deactivation of the ipsilateral sensorimotor cortex occurs during hand movement in healthy subjects [17]. Impaired deactivation of ipsilateral motor areas in MS patients during active hand movement has been recently described and interpreted as the result of reduced transcallosal inhibition [18], [19].

Furthermore, several brain areas in healthy humans, including parietal cortex, posterior cingulate cortex and mesial temporal cortex, i.e. areas of the default mode network (DMN), are more active during rest than during cognitive tasks [20]–[22]. The default mode areas are believed to be related to human higher-order cognitive and integrative functions and their deactivation increases with task difficulty [21]. Deactivation in these areas has also been reported during voluntary movement of the tongue and of the hand [18], [23], [24]. Little information exists about deactivation of the DMN during a motor task in MS.

In this study we decided to use a passive sensorimotor task, which so far has been applied, to our knowledge, in only two studies investigating the motor function in MS [25], [26]. Regardless of clear differences between an active, voluntary movement and a passive, non-voluntary movement, these two kinds of movement share similar higher motor control networks in normal subjects, in both location and extent [27]. A passive task is more suited to study motor function in patients since it is not influenced by individual performance and can be used to overcome high levels of disability as well as reduce inter-subject variability.

The aim of our study was to investigate fMRI changes during a passive sensorimotor task in MS patients with varying degrees of disability and tissue damage, with respect to healthy subjects.

We hypothesised that, despite the lack of volitional control in the passive hand movement task, relevant changes would occur in both cortical activation and deactivation in MS patients to reflect the degree of brain tissue damage and clinical disability.

## Materials and Methods

### Participants

We studied MS patients as diagnosed according to the McDonald criteria [28], recruited from the MS centre at our institution and affected by either the relapsing remitting (RRMS) or the secondary progressive (SPMS) form. The inclusion criteria were the following: age between 18 and 60 years; right-handed; previous recent evaluation of the Expanded Disability Status Scale (EDSS) score (less than 30 days); clinically stable disease for at least 30 days prior to study entry; no corticosteroid therapy 30 days prior to inclusion in the study; no disease-modifying therapy changes in the 6 months prior to enrolment in the study; no history of epilepsy or alcohol or substance abuse and no major medical illness; no psychiatric or cognitive impairment that precluded safe participation in the study; no concomitant therapy with antidepressant or psychoactive drugs; no female patient being pregnant, lactating or planning pregnancy during the course of the study.

According to the clinical form, we recruited 13 RRMS and 18 SPMS patients. We also included 15 right-handed healthy subjects who constituted the control group (HC). Healthy controls were selected to yield a mean age value between the mean age of the two patient groups and not to be significantly different from either.

All the participants gave their written informed consent to the study, which was approved by the Ethics Committee of the Sapienza University of Rome.

### MRI Procedures

FMRI scans were performed on a 1.5 T magnet scanner (Philips Gyroscan NT 15, Netherlands) with echo planar capabilities and a head volume radiofrequency coil. Each subject lay supine in the scanner with eyes closed. Head movements were minimized with foam padding and a restraining strap. Slice orientation parallel to the bi-commissural plane was assured by acquiring a multiplanar T1-weighted localizer at the beginning of each study. BOLD contrast was obtained using echo planar T2*-weighted imaging with the following parameters: TR/TE 3000/50 ms, 90° flip angle, one excitation, matrix size 64×64, field of view 24 cm x 24 cm, slice thickness 5 mm, gap 0 mm, 25 axial slices. Slice acquisition was ascending and we collected 145 volumes for each study.

Conventional proton density- and T2-weighted spin echo images (TR 2000 ms, TE 20/90 ms, matrix size 256×256, field of view 24 cm×24 cm, slice thickness 3 mm, gap 0 mm, 48 axial slices), and T1-weighted images (TR 600 ms, TE 15 ms, matrix size 256×256, field of view 24 cm x 24 cm, slice thickness 3 mm, gap 0, 48 axial slices), before and after the injection of a double dose (0.2 mg/kg) of gadolinium-diethylenetriamine penta-acetic acid (gd-DTPA), were acquired, in an ascending slice order.

### Motor Paradigm

During fMRI scanning, patients underwent passive right-hand movements according to a block design (6 blocks of rest alternated with 6 blocks of movement, for the duration of 21 seconds each). Immediately before undergoing the fMRI study, MS patients and HC were presented the task that was to be performed inside the magnet room. Passive movement consisted of simultaneous four-finger flexo-extension of the metacarpo-phalangeal joints executed by an operator (ET), at a rate of 1 Hz, guided by an acoustic cue; patients held their arms extended along their body, in a way that the operator could easily reach their right hand just outside the bore’s opening. They were instructed to close their eyes, relax both upper limbs and not to participate in the movement at all.

### MRI Data Analysis

Hyperintense T2 lesion volume (T2-LV) and hypointense (T1-LV) was calculated in each patient using the specialized medical image analysis software pack Jim 4.0 (Xinapse System, Leicester, England) with a semi-automated contouring technique, on the proton density and the T1-weighted image respectively, confirming the corresponding lesion in the T2-weighted image. [29].

FMRI data were analysed using a dedicated software and general linear model statistics (SPM8, Wellcome Department of Cognitive Neurology, England). The first 5 volumes were discarded in order to obtain BOLD steady state. For each study, image pre-processing included realignment, normalization and spatial smoothing using a Gaussian kernel of 8 mm. Images were then analysed using a two-level random-effect approach.

At the first level, time series of functional MR images obtained from each participant were analysed separately. The effects of experimental paradigm were estimated on a voxel-by-voxel basis using the principles of the general linear model, extended to allow the analysis of fMRI data as a time series [30]. The data regarding each subject were modelled using a boxcar design, convolved with the canonical hemodynamic response function which was chosen to represent the relationship between neuronal activation and blood-flow changes. Statistical significance of signal changes related to hand movement were determined on a voxel-by-voxel basis using a t-statistic, which was then transformed into a normal distribution. Two contrast images were created for each subject: 1.Task-related activation during passive movement (Task>Rest); 2.Task-related deactivation during passive movement (Rest>Task). The activation and deactivation maps were verified in all subjects before the second level step started.

At the second level, individual contrast images were entered into an one-way ANOVA design under SPM8, including age and gender as nuisance covariates to control for these variables within the study population. After checking for a significant main effect of group, two masks were created at p<0.05 family-wise error (FWE) corrected, including the average effect of condition for activation and deactivation, respectively. These masks were derived from thresholding the F-statistic image using FWE correction at p = 0.05, and were used to identify significant clusters of activation and deactivation in the post-hoc between-groups and within-groups comparisons by constraining the analysis to areas within the masks.

Regions of significant activation or deactivation were identified by using the SPM8 tool “AAL”, which uses the Montreal Neurological Institute (MNI) spatial coordinates of a standard labelled brain atlas to attribute neuroanatomical localization for these regions [31].

To evaluate differences in cortical activity between groups in both activation and deactivation, we created plots of contrast estimates, centred on the voxels of global maxima in the average effect of condition F-maps.

To test the relationship between areas of activation and deactivation within subjects across the three groups, we performed a correlation analysis between clusters of maximal activation in the left sensorimotor cortex and those of maximal deactivation in the posterior cortical areas by using the Marsbar toolbox of SPM8 for region of interest analysis [32].

Lastly, a correlation analysis to assess the influence of tissue damage on brain activity was applied to the whole patient population and separately to both patient groups, by entering T2-LV as a covariate of interest in a one-sample model. Correlations of T2-LV with activation or deactivation areas were restricted to voxels displaying significant activation or deactivation by applying the above-mentioned masks. Same correlation analysis was replicated using T1-LV.

### Statistics

Differences in clinical and radiological parameters between RRMS and SPMS were examined by unpaired t-test by using PASW Statistics 18, Release Version 18.0.0 (SPSS, Inc., 2009, Chicago,IL, www.spss.com). Statistical threshold was set at p<0.05.

## Results

### Participants

The demographic and clinical characteristics of the two patient groups (RRMS and SPMS) and the healthy volunteers (HC) are described in **Table 1**. RRMS and SPMS groups were significantly different as regards age, disease duration and EDSS; both T2-LV and T1-LV were lower, though not significantly, in the RRMS group than in the SPMS group. No patient showed any gd-DTPA enhancing lesions.

**Table 1:** Demographic and clinical/radiological characteristics of 31 MS patients (13 RRMS, 18 SPMS) and 15 HC enrolled in the study.

	RRMS	SPMS	HC
	n = 13	n = 18	n = 15
**Gender, F/M**	7/6	12/6	8/7
**Age (years)**	37.9 (10.4)*	49.8 (6.4)	41.7 (9.0)
**mean (SD)**			
**MS duration (years)**	7.6 (5.8)*	21.9 (8.6)	-
**mean (SD)**			
**EDSS score**	1.5. [1.0–3.0]	6.0 [6.0–6.5]	-
**median [range]**			
**T2-LV (mmˆ3)**	6297 (5906)	10004 (9837)	
**mean (SD)**			
**T1-LV (mmˆ3)**	1731 (2335)	2908 (4904)	-
**mean (SD)**			

EDSS = Expanded Disability Status Scales; T2-LV = T2-hyperintense lesion volume; F = Females M = Males; HC = Healthy Controls; RRMS = Relapsing Remitting MS patients; SPMS = Secondary Progressive MS patients; SD = Standard Deviation.

* significantly lower than SPMS group by unpaired t-test (p<0.05).

### Functional Imaging Analysis

Average effect of condition F test showed significant clusters of activation in the contralateral sensorimotor cortex, in the ipsilateral sensorimotor cortex, in the supplementary motor area bilaterally and in the ipsilateral cerebellum as well clusters of deactivation in the parieto-occipital cortex bilaterally, in the right parahippocampal region and in the ipsilateral motor cortex.

### Within-group Analysis

**Figure 1:**
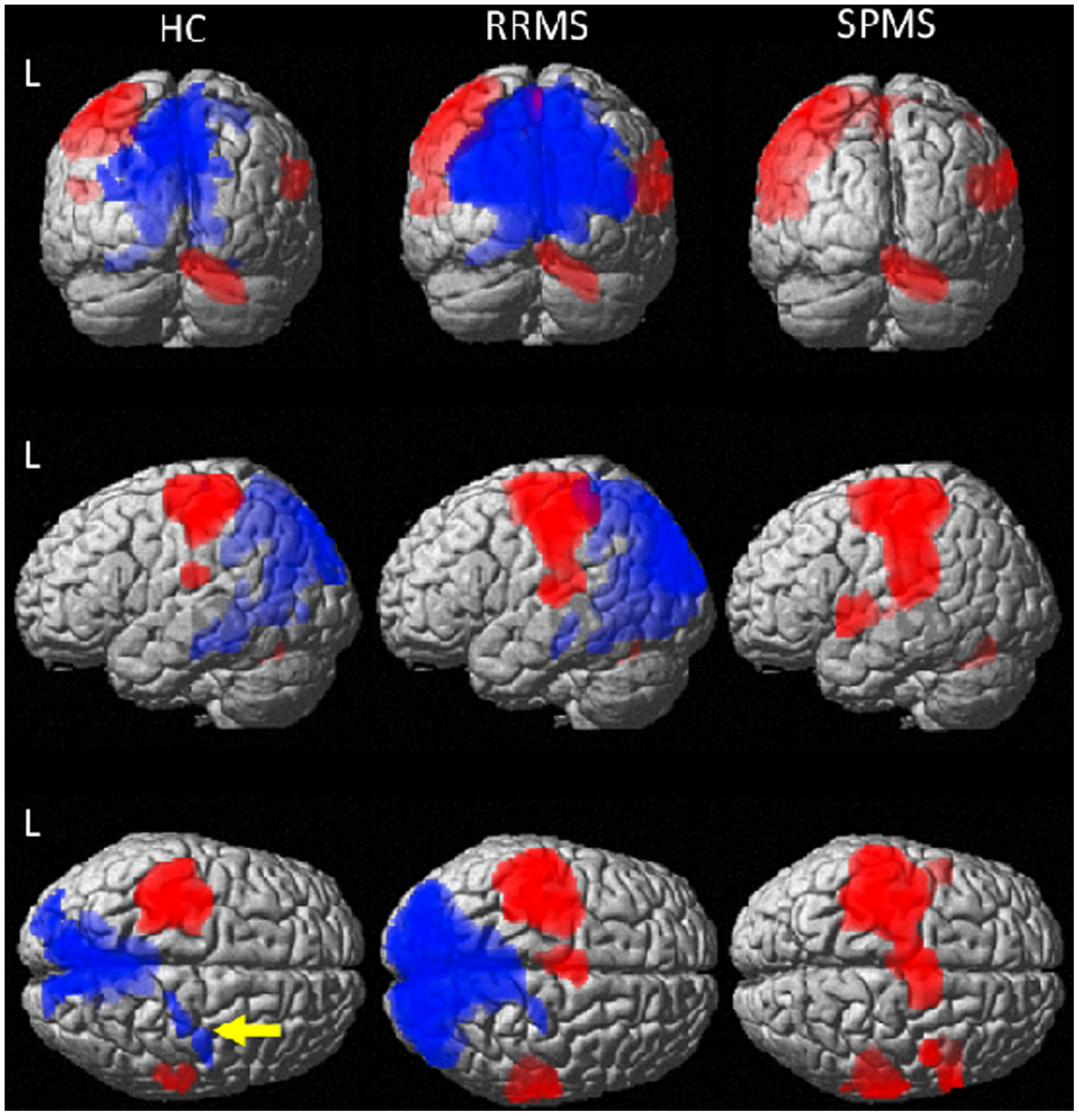
Brain regions that showed significant activation (red) and deactivation (blue) during passive right hand movement in three groups of subjects: healthy controls (HC), relapsing-remitting MS patients (RRMS) and secondary progressive MS patients (SPMS). Maps obtained by one sample t-test, corrected for age and gender at p<0.05 FWE (cluster level) are superimposed to a rendered T1 brain image. Cluster maxima for activation (red) included cerebellum and vermis, left pre- and postcentral gyri and inferior parietal lobule in all groups. In both RRMS and SPMS groups the left sensorimotor activation extended to include the adjacent parietal and temporal cortex and additional foci were observed in the supplementary motor area bilaterally and in the right (ipsilateral) cortical areas. In SPMS patients, activation was even more extended and involved additional cortical foci, i.e. the middle frontal gyrus, insula and temporal pole bilaterally. Significant deactivation (blue) was observed in the ipsilateral motor areas (arrow) in HC but not in either MS group. Deactivation in cortical areas outside the motor system, i.e. cuneus, precuneus and temporal areas, was present in HC and extended in RRMS MS patients. No foci of deactivation were observed in the SPMS group at this level of significance (for more details see Tables 2 and 3).

In the HC group, significant activation during passive right-hand movement was observed in the right cerebellum and vermis, in the left pre- and postcentral gyri and in the left rolandic operculum and supramarginal gyri (**Figure 1**, **Table 2**).

**Table 2:** Brain regions that showed significant activation during passive right hand movement within each of the 3 study groups.

Group	Peak MNI coordinate (x y z)			Peak Z-score	Side	Brain regions included in clusters/sublcusters	Number of voxels in Cluster
**HC**	14	−54	−22	6.98	R	Cerebellum/Vermis	1031
	−34	−32	60	6.30	L	Pre−/Postcentral gyrus	2699
	−42	−24	20	4.26	L	Rolandic Operculum	1897
					L	Postcentral gyrus	
					L	Supramarginal gyrus	
**RRMS**	−32	−20	66	6.83	L	Pre−/Postcentral gyrus	4465
	16	−54	−24	6.01	R	Cerebellum/Vermis	816
	−4	−8	54	5.22	Bi	Supplementary Motor Area	619
	56	−18	20	4.92	R	Superior Temporal Gyrus	1184
					R	Supramarginal gyrus	
					R	Rolandic Operculum	
					R	Postcentral gyrus	
**SPMS**	−36	−30	60	6.56	L	Pre−/Postcentral gyrus	7326
					L	Supramarginal gyrus	
					L	Inferior Parietal gyrus	
					Bi	Supplementary Motor area	
					L	Superior Temporal gyrus	
	20	−54	−24	6.09	R	Cerebellum/Vermis	1479
	48	0	52	5.40	R	Middle Frontal gyrus	154
					R	Precentral gyrus	
	56	12	2	4.85	R	Inferior Frontal operculum	989
					R	Insula	
					R	Rolandic Operculum	
	56	−22	18	4.30	R	Supramarginal gyrus	1141
					R	Superior Temporal gyrus	
					R	Rolandic Operculum	
					R	Postcentral gyrus	
	−52	6	2	4.28	L	Insula	575
					L	Rolandic Operculum	
					L	Superior Temporal gyrus	
					L	Temporal Pole	

L = left; R = Right; Bi = Bilateral; MNI = Montreal Neurological Institute.

HC = Healthy Controls; RRMS = Relapsing Remitting MS patients; SPMS = Secondary Progressive MS patients.

Results obtained from a one-sample t-test after correcting for age, *p*<0.05 FWE corrected.

In patients, significant activation areas extended further to include adjacent frontal and parietal cortices, supplementary motor area (SMA) bilaterally and superior temporal gyri in the contralateral hemisphere, as well as a series of significant foci in the ipsilateral hemisphere. This extended activation was more evident in SPMS patients (**Figure 1** **,** **Table 2**).

The deactivation pattern in HC showed significant foci located in the ipsilateral motor areas and in cerebral areas outside the motor system, including posterior portions of temporal, parietal and occipital lobes bilaterally.

Neither patient group showed any significant deactivation of the ipsilateral motor cortex. The extent of deactivation in the posterior parietal and occipital cortex was greater in the RRMS group than in HC (**Figure 1** **,** **Table 3**). The SPMS group did not reveal any significant cluster of deactivation in posterior areas at this level of significance (**Figure 1** **,** **Table 3**). However, at a lower threshold (p<0.001 uncorrected) the majority of the posterior clusters of deactivation also appeared in the SPMS group.

**Table 3:** Brain regions that showed significant deactivation during passive right hand movement within each of the three groups.

Group	Peak MNI coordinates(x y z)			Peak Z	Side	Brain regions included in clusters/sublcusters	Number of voxels in Cluster
**HC**	22	−16	−28	5.03	R	Hippocampus/Parahippocampal gyrus	310
	−10	−80	40	4.96	Bi	Cuneous/Precuneus	7074
	36	−10	34	4.77	R	Precentral gyrus	395
					R	Postcentral gyrus	
**RRMS**	−18	−82	16	6.22	Bi	Cuneous/Precuneus	18980
					Bi	Middle/Superior Occipital gyri	
					Bi	Lingual gyri	
	20	−22	−22	3.98	R	Hippocampus/Parahippocampal gyrus	175
	−26	−32	−8	3.90	L	Hippocampus/Parahippocampal gyrus	213
**SPMS**	n.s.						

L = left; R = Right; Bi = Bilateral; MNI = Montreal Neurological Institute.

HC = Healthy Controls; RRMS = Relapsing Remitting MS patients; SPMS = Secondary Progressive MS patients; n.s. = not statistically significant.

Results obtained from a one-sample t-test after correcting for age, p<0.05 FWE corrected.

### Between-group Analysis

**Figure 2:**
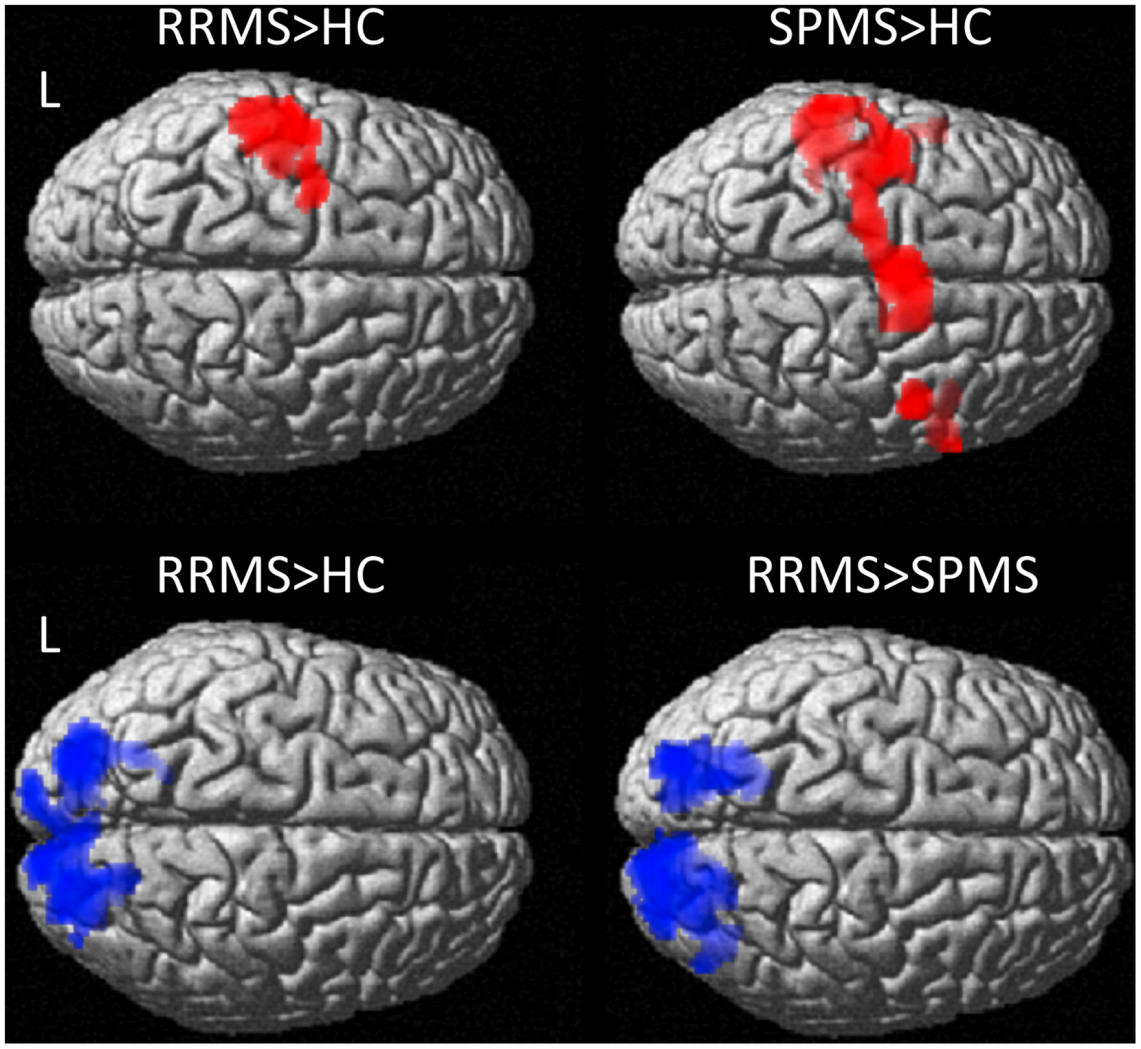
Brain regions that showed significant differences between groups in activation (red) and deactivation (blue) during passive right hand movement. Maps obtained by two sample t-test, corrected for age and gender at p<0.05 FWE (cluster level) are superimposed to a rendered T1 brain image. HC = healthy controls, RRMS = relapsing-remitting MS patients; SPMS = secondary progressive MS patients.

Significant differences in cortical brain activity were observed between the three groups during passive hand movements (**Figure 2**). RRMS patients showed greater activation than HC in the left pre- and postcentral, inferior parietal gyrus and supramarginal gyri. SPMS patients displayed greater activity than HC in the left pre- and postcentral, middle frontal, supramarginal, superior and middle temporal gyri, rolandic operculum, in the SMA bilaterally, and in the right precentral gyrus.

RRMS patients displayed greater deactivation than HC and SPMS in cuneous/precuneus bilaterally (**Figure 2**).

**Figure 3:**
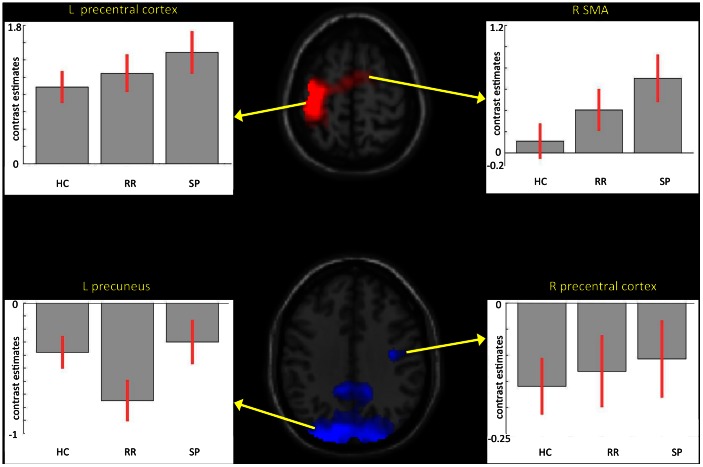
Plots of contrast estimates of brain activity in three groups of subjects: healthy controls (HC), relapsing-remitting (RRMS) MS patients and secondary progressive (SPMS) MS patients, centered on the activity peaks of the average effect of conditions (activation in red, deactivation in blue) by ANOVA (p<0.05 FWE corrected at cluster level). Plots show that significant differences in brain activation between groups in the left precentral (cluster maxima at −36, −30, 60) cortex and in the left supplementary motor area (SMA) (cluster maxima at 4, −6, 66) was due to increased activation in SPMS than in RRMS and in RRMS than in HC (top). Plots show that significant differences in brain deactivation between groups in the left precuneus (cluster maxima at −24, −90, 34) was due to greater deactivation in the RRMS than in the other two groups whereas in the ipsilateral motor cortex (cluster maxima at 36, −12, 36), deactivation was greater in HC rather than in patients (bottom). (All coordinates refer to the MNI standard brain).

Plots of contrast estimates, centred on the global maxima voxel in the average effect of condition F-maps, revealed the amount of brain activity in the three groups; in the left precentral gyri and right supplementary, activation increased progressively from HC to RRMS and from RRMS to SPMS; in the precuneus, the greatest deactivation was found in the RRMS; finally, in the ipsilateral precentral cortex, deactivation decreased progressively from HC to RRMS and from RRMS to SPMS (**Figure 3**).

### Activation/Deactivation Correlation

In the activation/deactivation correlation analysis three healthy subjects, two RRMS and eight SPMS patients were not included because of the lack of significant clusters in the left sensorimotor cortex and/or in the posterior cortical areas overlapping the DMN.

**Figure 4:**
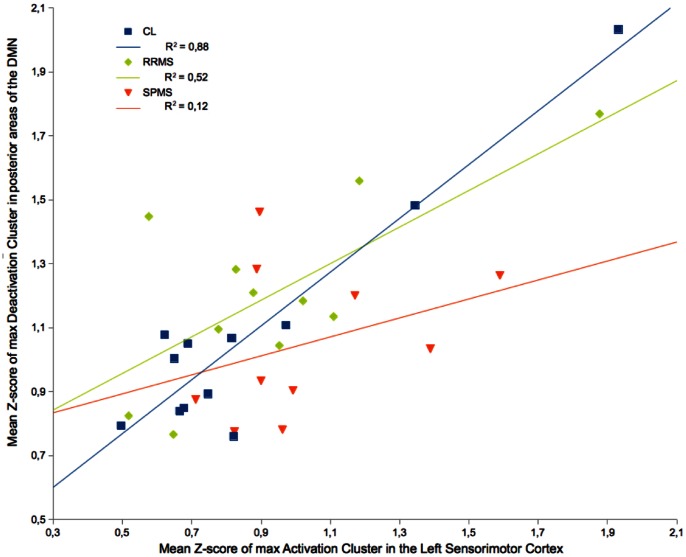
Correlation between mean Z-score of maximum activation cluster in left sensorimotor cortex and mean Z-score of maximum deactivation in the posterior corical areas overlapping the Default Mode Network (DMN). R^2^ values and corresponding linear regression estimate lines for healthy controls (HC) in blue, relapsing-remitting MS patients (RRMS) in light green and secondary progressive (SPMS) in red, are reported. Significant correlation was found in HC (R^2^ = 0.88, p<0.001), in RRMS (R^2^ = 0.52, p<0.01), but not in SPMS.

In the remaining subjects, the analysis showed that activation in the left sensorimotor cortex was significantly correlated with posterior deactivation in the control group (p<0.001) and in the RRMS group (p<0.01). No correlation between the amount of activation and that of deactivation was found in the SPMS group (Figure 4).

### Correlation with Tissue Damage

**Figure 5:**
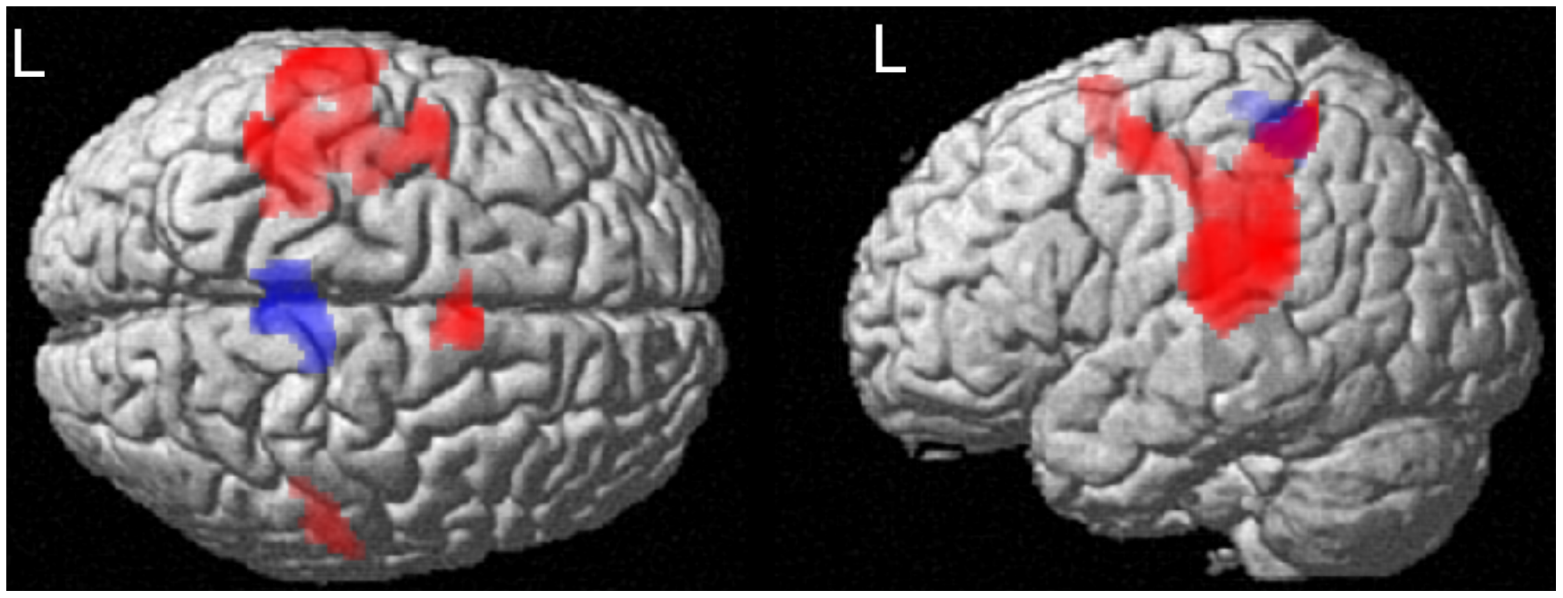
Foci of activation (red) and deactivation (blue) that showed a positive and negative correlation, respectively (p<0.05 FWE corrected at cluster level) with T2-LV, superimposed to a rendered T1 brain image. The T2-LV positively correlated with activation in clusters located in the left supramarginal gyrus (Z = 5.85, MNI coordinates: −52, −42, 24), right SMA (Z = 5.04, MNI coordinates: 4, 8, 56) and right superior temporal gyrus (Z = 4.18, MNI coordinates: 56, −30, 16). The T2-LV negatively correlated (p<0.05 FWE corrected at cluster level) with deactivation in the precuneus and paracentral lobule bilaterally with the activity peak centered in the right paracentral lobule (Z = 3.90, MNI coordinates: 2, −34, 58).

Activation and deactivation foci that were significantly correlated with either T2-LV or T1-LV are shown in **Figure 5**. The T2-LV positively correlated (p<0.05 FWE corrected at cluster level) with activation centred around the left supramarginal gyrus (Z = 5.85, MNI coordinates: −52, −42, 24), right SMA (Z = 5.04, MNI coordinates: 4, 8, 56) and right superior temporal gyrus (Z = 4.18, MNI coordinates: 56, −30, 16) in the whole patient sample; the T2-LV negatively correlated (p<0.05 FWE corrected at cluster level) with deactivation in the precuneus and paracentral lobule bilaterally (Z = 3.90, MNI coordinates: 2, −34, 58).

T1-LV significantly correlated with activation centred around the left supramarginal gyrus (Z = 5.03, MNI coordinates: −50, −42, 24), overlapping the results of T2-LV correlation. No significant correlation was found between T1-LV and deactivation.

When considering the two patient groups separately, significant correlation between T2–LV and activation in foci localised in the left parietal cortex persisted only in the SPMS group.

Correlation between T1-LV and activation in the RRMS group and between T1-LV and deactivation in both groups did not reach statistical significance, likely because of the small sample sizes.

## Discussion

Our results show that, in MS patients, passive hand movements are associated with an increased activation of contralateral and ipsilateral cortical sensorimotor regions. The extent of this activation increases across disease forms, i.e., from RRMS to SPMS, and correlates with the amount of tissue damage. Passive hand movements are also associated with altered deactivation of sensorimotor and posterior parietal and occipital areas. Deactivation in the ipsilateral sensorimotor cortex is reduced in patients, compared to healthy volunteers, paralleling their increased ipsilateral sensorimotor activation. Our study also reveals the dissociation between activation in sensorimotor areas and deactivation in posterior DMN regions in MS patients with higher disease burden, suggesting an altered reciprocal modulation of brain networks as a result of increasing MS damage.

While results on passive movement extended notions of previous studies on brain activation during volitional movements [25], [26], our finding of changes in deactivation outside the sensorimotor network during passive hand movements in MS patients is quite novel.

### Activation Patterns

In our study, passive hand movements in patients were associated with a pattern of activation that included the contralateral sensorimotor cortex and ipsilateral cerebellum. This pattern was more widespread in MS patients than in HC and extended to frontal, temporal and parietal cortices adjacent to the contralateral sensorimotor cortex, the SMA and the sensorimotor areas of the ipsilateral hemisphere. This extended activation was more evident in patients who showed more severe clinical disability and tissue damage, i.e. the SPMS group.

While an increased extent of activations from healthy controls to RRMS patients and from RRMS to SPMS patients has been previously described across MS forms during active hand movement [7], here we provide the first evidence that this also holds true for passive movements. The increase in ipsilateral cortical activation during passive hand and foot movements, described in MS [25], [26], was related to brain tissue damage and clinical disability [25]. This suggests that activation of additional cortical areas is independent of the perceived complexity of the task or of the amount of effort made to perform the task. Those studies argued that patients undergo functional reorganisation of their brain networks, independently from volitional control or ability. Our findings support this concept and extend its validity across the RRMS and SPMS forms of MS.

Our study revealed a positive correlation between lesion volume and activation of regions involved in higher motor control and sensorimotor integration such as the left supramarginal gyrus, right SMA and right superior temporal gyrus. The involvement of these regions as disease burden increases may represent adaptive functional reorganisation, as suggested by previous fMRI studies [3], [5], [9]–[12], [16]. Alternatively, increased cortical excitability due to decreased activity of inhibitory intracortical circuits in the SPMS form, as demonstrated by TMS studies [33], may account for the increased cortical activation observed in SPMS patients.

### Deactivation Patterns

In healthy subjects, passive hand movements were associated with deactivation of ipsilateral sensorimotor regions, as well as posterior parietal, occipital and temporal areas bilaterally.

In MS patients, deactivation of the ipsilateral sensorimotor cortex was reduced when compared to healthy volunteers, in agreement with the findings described by Manson in RRMS or SPMS [18]. An impaired interhemispheric physiological inhibition [34],19] could explain this finding. Since deactivation in ipsilateral motor areas mainly depends on decreased transcallosal inhibition, our findings suggest a manifestation of damage to the corpus callosum [35]. Alternatively, decreased deactivation in the ipsilateral sensorimotor cortex could be the result of a greater activity of this region at rest in the patients. The hypothesis of an increased resting activity in this region is supportive of the adaptive role of ipsilateral sensorimotor areas in functional recovery after MS damage [12], [16].

In both patients and controls, passive task-related deactivation involved posterior parietal, occipital and temporal areas bilaterally, most of which are also observed during cognitive tasks and contribute to the DMN [20]–[22]. These results suggest that modulation of brain’s functional state occurs when the resting condition is interrupted, independently of the task’s type or difficulty.

The DMN deactivation during cognitive tasks increases with task difficulty [21], paralleling task-induced activation [36]. During passive sensory tasks, an increased DMN deactivation accompanies a greater task-related activation [37].

Clusters of posterior deactivation also included areas not belonging to the DMN, i.e, the visual cortex. In this case, deactivation most likely reflects cross-modal sensory inhibition [38].

Finally, we found a negative correlation between increasing lesion burden and the extent of deactivation in the paracentral lobule and precuneus bilaterally, i.e., increasing damage was associated with reduced deactivation, suggesting that progression of the disease and accumulation of tissue damage alter the ability of the DMN areas to counter-balance the activity of task-related areas.

### Activation/Deactivation Balance

In our study a significant correlation was found between the amount of activation in the left sensorimotor cortex and that of deactivation in the posterior cortical areas overlapping the DMN in healthy subjects and in RRMS patients. This correlation was not present in SPMS patients.

These findings support the concept of a inter-network relationship between the sensorimotor network and the DMN, which is impaired in the more advanced phases of the disease.

In RRMS, both activation of sensorimotor areas and deactivation of DMN were increased with respect to healthy subjects, indicating that the activation/deactivation balance was maintained. since increased DMN deactivation was associated with the increased sensorimotor activation; this finding may be interpreted as a task-dependent functional modulation, which could facilitate motor circuit engagement during task performance.

Our finding of an altered activation/deactivation interplay in SPMS patients may represent an altered mechanism of reciprocal modulation between brain areas, which may occur in SPMS as a consequence of a higher disconnection, combined with insufficient brain plasticity adaptation.

The significant correlation between the lesion burden and the amount of activation and deactivation in the whole patient sample further supports this interpretation.

### Limits

We cannot exclude that those differences in demographic and physiological factors between patient groups may contribute to our results. We tried to control for the effect of some of these factors (age, gender) in our analysis model. Considering that we used a passive task, we expected that the effects of task induced variations of physiological parameters (cardiovascular and respiratory) on the generation of BOLD signal would be less prominent and thus would not invalidate our results. We also took a correlative approach, along with the investigation of the between-group differences, when exploring the relationship between disease burden and functional responses. This approach should be less prone to suffer from the typical confounding effects of fMRI studies [39].

### Conclusions

Overall, our study, using a passive task to explore functional reorganisation with increasing MS disease burden, suggests that the balance between activation and deactivation, reflecting the functional integration of brain networks, is affected in patients. The study of the functional expressions of specific areas belonging to the sensorimotor and DMN networks may thus provide targets for interventional studies aiming to rebalance brain function.
